# Gender and age-specific aspects of awareness and knowledge in basic life support

**DOI:** 10.1371/journal.pone.0198918

**Published:** 2018-06-12

**Authors:** Mario Krammel, Sebastian Schnaubelt, David Weidenauer, Markus Winnisch, Matthias Steininger, Jakob Eichelter, Thomas Hamp, Raphael van Tulder, Patrick Sulzgruber

**Affiliations:** 1 Department of Anesthesiology, Intensive Care and Pain Medicine, Medical University of Vienna, Vienna, Austria; 2 Emergency Medical Service Vienna, Vienna, Austria; 3 Austrian Cardiac Arrest Awareness Association – PULS, Vienna, Austria; 4 Department of Emergency Medicine, Medical University of Vienna, Vienna, Austria; 5 Department of Medicine II, Division of Cardiology, Medical University of Vienna, Vienna, Austria; 6 Department of Trauma Surgery, Medical University of Vienna, Vienna, Austria; 7 Department of Internal Medicine I, Division of Cardiology, Karl Landsteiner University of Health Sciences, Krems, Austria; TNO, NETHERLANDS

## Abstract

**Background:**

The ‘chain of survival’—including early call for help, early cardiopulmonary resuscitation (CPR) and early defibrillation—represents the most beneficial approach for favourable patient outcome after out-of-hospital cardiac arrest (OHCA). Despite increasing numbers of publicly accessible automated external defibrillators (AED) and interventions to increase public awareness for basic life support (BLS), the number of their use in real-life emergency situations remains low.

**Methods:**

In this prospective population-based cross-sectional study, a total of 501 registered inhabitants of Vienna (Austria) were randomly approached via telephone calls between 08/2014 and 09/2014 and invited to answer a standardized questionnaire in order to identify public knowledge and awareness of BLS and AED-use.

**Results:**

We found that more than 52 percent of participants would presume OHCA correctly and would properly initiate BLS attempts. Of alarming importance, only 33 percent reported that they would be willing to perform CPR and 50 percent would use an AED device. There was a significantly lower willingness to initiate BLS attempts (male: 40% vs. female: 25%; OR: 2.03 [95%CI: 1.39–2.98]; p<0.001) and to use an AED device (male: 58% vs. female: 44%; OR: 1.76 [95%CI: 1.26–2.53]; p = 0.002) in questioned female individuals compared to their male counterparts. Interestingly, we observed a strongly decreasing level of knowledge and willingness for BLS attempts (-14%; OR: 0.72 [95%CI: 0.57–0.92]; p = 0.027) and AED-use (-19%; OR: 0.68 [95%CI: 0.54–0.85]; p = 0.001) with increasing age.

**Conclusion:**

We found an overall poor knowledge and awareness concerning BLS and the use of AEDs among the Viennese population. Both female and elderly participants reported the lowest willingness to perform BLS and use an AED in case of OHCA. Specially tailored programs to increase awareness and willingness among both the female and elderly community need to be considered for future educational interventions.

## Introduction

With more than 35/100.000 citizens affected per year independently in Europe, out-of-hospital cardiac arrest (OHCA) shows a high mortality and morbidity and renders poor survival rates of less than 20%. [1] A ‘chain of survival’—including early call for help, early cardiopulmonary resuscitation and early defibrillation—represents the most beneficial approach for favourable patient outcome after this fatal cardiac event. [2]

Times from an emergency call to the arrival of the emergency medical service team at the scene (= response time) show large regional differences between countries and among both rural and urban areas. However, in most urban communities within the western society, median response times of 5–8 minutes can be achieved. [3–4] Additionally, the time until the first defibrillation by healthcare professionals is often delayed up to 11 minutes after the emergency call. [5–6] Considering a decreasing chance of survival of 10% per minute delay of basic life support (BLS) and public access automated external defibrillation (AED), only a small fraction of patients suffering OHCA remains potentially salvageable. [7] Recent studies were able to demonstrate that the use of public AED devices in addition to bystander cardiopulmonary resuscitation (CPR) was able to double survival rates in patients presenting with an initially shockable ECG rhythm. [8] However, of alarming interest, Nürnberger and co-workers revealed that a public AED was only administered to 4% of all OHCA victims, reflecting a dramatic underuse in the Viennese community. [9] Since there has been a significant increase in the number of public AED devices that are easily identifiable in public places, reasons for this strong underuse of AED might be based on insufficient knowledge, awareness or willingness in the use of the respective devices. In this regard, data on the awareness and knowledge among the Viennese population remain unknown, but seem of major importance for further strategies in the implementation of public AED networks und educational strategies.

Therefore, we aimed to elucidate aspects of the awareness, the knowledge and willingness in both BLS and the use of public AED devices in Vienna.

## Methods

### Study setting and population

Vienna (Austria) represents one of the largest catchment areas in Europe, covering nearly 3 million citizens with a population density of 4502 persons per square kilometer. More than 830 public AED devices are available in selected public locations across the city, with a majority accessible 24 hours, 7 days a week. A standardized logo—green background, with a white heart and lightning symbol—is clearly advertised in accordance to recommendations of the International Liaison Committee on Resuscitation (ILCOR), so that the respective locations for public AEDs can be easily identified as such for BLS/AED providers.

Considering the general knowledge of BLS and the use of an AED in the general Austrian population, the completion of a first-aid course is complementary at several points—for example obtaining a driver’s license—in Austria and is regulated by law in terms of content (must include BLS and AED training) and duration (16 hours). Therefore, most inhabitants of Austria have completed a standardized 16-hour first-aid course that includes training in BLS and AED use as recommended by the European Resuscitation Council. [10]

### Study design

In this prospective population-based cross-sectional study, a total of 501 residents of Vienna were randomly approached via telephone call by specially trained personnel of the Institute for Empiric Social-Science (Institut für Empirische Sozialforschung, IFES, Vienna, Austria) and invited to answer a standardized questionnaire. Selection of participants was conducted via a computer-based randomization of all registered telephone numbers (including mobile phone numbers) of Viennese inhabitants via IFES. The population of Vienna was chosen for this survey based on its homogenous characteristics and due to reflecting the general population of the ‘western society’ from an Austrian perspective.

In order to obtain a true cross section of the Viennese population, no specific exclusion criteria were applied. For inclusion in the study, participants needed to be registered inhabitants of the city of Vienna (Austria). Individuals that agreed to participate and that were thought to be fit in age and comprehension to clearly answer the given questions (speak German language) were confronted with the structured multiple-choice questionnaire that aimed at identifying aspects of knowledge and awareness concerning BLS and the use of public AEDs. Using the afore mentioned randomization-method for selection of participants, we were able to enroll an overall balanced study population considering gender (male: 42.5%) and age (<45a: 31.8%; 45-65a: 37.9%; >65a: 30.3%), and subsequently minimalized the risk of bias und unknown confounding.

The questionnaire was similar to two surveys on first-aid in Melbourne and Southampton and is evident as supplementary file (see S1 and S2 Files). [11–12] Enrollment of participants was conducted between 08/2014 and 09/2014. Assessing knowledge of BLS and AED-use, participants were confronted with a standardized questionnaire including four possible answers to the respective question (including only one right answer) that needed to be picked. Moreover several questions were designed in a yes/no-style that also allowed ‘not sure’ as an exit. Interestingly, none of the participants chose this possible answer. A detailed copy of both the used questionnaire and an English version are evident as supplementary information (see S1 and S2 Files).

The local Ethics Committee of the Medical University of Vienna (Austria) reviewed the study-protocol of the present analysis and confirmed that surveys meeting audit criteria are not eligible for ethics peer review and do not require ethics approval. However, all participants gave oral informed consent prior to study enrollment due to minimal risk of study inclusion.

### Data processing and statistical analysis

Completed anonymized questionnaires were reviewed by trained chart reviewers and subsequently inserted into a predefined record abstraction form for further analysis. Discrete data are presented as counts and percentages and were compared using chi-square test. A logistic regression model for the association of age and gender on the awareness and knowledge of BLS and AED was performed. Data were presented as odds ratio (OR) and the respective 95% confidence interval (CI). For statistical analysis SPSS 21.0 (IBM, USA) was used. Considering gender and age-specific incidence rates of awareness and knowledge on BLS (40% vs. 25%; 40% vs. 26%) and AED use (58% vs 44%; 57% vs. 37%), a sample size of 500 enrolled participants offers a statistical power of greater 0.8 (alpha: 0.05). A p-value < 0.05 (two-tailed) was defined as statistically significant.

## Results

Out of 501 contacted persons 213 (43%) were male and 288 (58%) female. The age distribution proved to be balanced comparing age groups (<45 years: 159 [32%]; 45–65 years: 190 [38%]; >65years: 152 [30%]. A first-aid course had been completed by 408 (81%) participants.

### Knowledge on cardiac arrest and basic life support

A total of 326 participants (65%) found that BLS attempts by laypersons in case of cardiac arrest are very important for patient outcome. Additionally, 52% (n = 216) of all contacted persons would control the patient´s breathing after being confronted with an unresponsive patient. More than 31% would place the respective patient in a recovery position without checking for breathing. Of alarming importance, while 58% (n = 291) of individuals reported that chest compressions are the most beneficial therapeutic approach in case of cardiac arrest, 29% indicated rescue breaths as the most promising technique for the survival of the patient. Of note, we found that only 33% (n = 166) of all participants are willing to perform CPR in case of witnessed cardiac arrest. (Table 1)

**Table 1:** Characteristics of the entire study population. Discrete data are presented as counts and percentages.

Participants’ knowledge and awareness on BLS and AED-use	
Thinks that CPR by layperson is important for survival, n (%)	326 (65)
Would check for breathing in case of unconsciousness, n (%)	261 (52)
Thinks that chest compressions are most beneficial for outcome, n (%)	291 (58)
Is willing to perform CPR, n (%)	166 (33)
Knows that the defibrillator can save the patients’ life with eclectic shocks, n (%)	486 (97)
Knows that everybody is allowed to use an AED in case of CA, n (%)	286 (57)
Knows the color of the standardized logo for AEDs, n (%)	251 (50)
Is willing to use an AED, n (%)	251 (50)

BLS = basic life support; AED = automated external defibrillator/defibrillation; CPR = cardiopulmonary resuscitation; CA = cardiac arrest.

### Knowledge on automated external defibrillator use

Within the entire study population nearly all participants (97%) correctly identified the defibrillator as the device “that is able to safe the patient´s life using an electric ‘shock’”. However, while 57% (n = 286) of individuals reported that everybody is allowed to use an AED, 21% assumed that only physicians are allowed to use a defibrillator. Additionally, only 50% (n = 251) of participants were able to name the color of the standardized logo for AEDs correctly. Moreover, only 50% (n = 251) of all contacted persons reported that they would be willing to use an AED device in case of witnessed cardiac arrest. (Table 1)

### Gender and age-specific aspects

Comparing male and female participants, we found similar results considering the knowledge of BLS and AED-use. However, female individuals reported a significantly lower willingness to initiate BLS attempts (male: 40% vs. female: 25%; OR: 2.03 [95%CI: 1.39–2.98]; p<0.001) and to use an AED device (male: 58% vs. female: 44%; OR: 1.76 [95%CI: 1.26–2.53]; p = 0.002). Moreover, after stratification into age-groups (<45years; 45-65years: >65years) we found that the youngest age group clearly demonstrated the best knowledge of BLS and AED-use and the highest willingness to perform critical care attempts in case of witnessed OHCA. In this regard, we observed a strongly decreasing willingness for BLS attempts (-14%; OR: 0.72 [95%CI: 0.57–0.92]; p = 0.027) and AED-use (-19%; OR: 0.68 [95%CI: 0.54–0.85]; p = 0.001) with increasing age. (Table 2)

**Table 2:** Participants’ knowledge and awareness on BLS and AED-use stratified by gender and age-groups. Discrete data are presented as counts and percentages and were compared using Chi-Square test. Logistic regression model for the association age, gender and first-aid education on the awareness and knowledge concerning BLS and AED use. Data are presented as odds ratio (OR) and the respective 95% confidence interval (CI).

Participants’ knowledge and awareness on BLS and AED-use stratified by gender and age-groups.						
**Gender**	**Male**	**Female**			**Crude OR (95%CI)**	p-value*
	**N = 213**	**N = 288**				
Thinks that CPR by laypersons is important for survival, n (%)	139 (65)	187 (65)			1.02 (0.70–1.47)	0.939
Would check for breathing in case of unconsciousness, n (%)	115 (54)	146 (52)			1.14 (0.80–1.63)	0.465
Thinks that chest compressions are most beneficial for outcome, n (%)	123 (57)	168 (58)			0.98 (0.68–1.39)	0.895
Is willing to perform CPR, n (%)	87 (40)	73 (25)			2.03 (1.39–2.98)	**<0.001**
Knows that the defibrillator can save the patient’s life with eclectic shocks, n (%)	213 (99)	271 (94)			6.62 (1.51–28.96)	**<0.001**
Knows that everybody is allowed to use an AED in case of CA, n (%)	117 (55)	142 (49)			1.25 (0.88–1.79)	0.213
Knows the colour of the standardized logo for AEDs, n (%)	122 (57)	129 (44)			1.65 (1.15–2.36)	**0.006**
Is willing to use an AED, n (%)	124 (58)	127 (44)			1.76 (1.26–2.53)	**0.002**
**Age Groups**	**<45 years**	**45-65years**	**>65years**	**p-value**		
	**N = 159**	**N = 190**	**N = 152**			
Thinks that CPR by laypersons is important for survival, n (%)	106 (67)	127 (67)	93 (61)	0.484	0.89 (0.70–1.12)	0.315
Would check for breathing in case of unconsciousness, n (%)	108 (68)	94 (50)	59 (39)	**<0.001**	0.55 (0.43–0.69)	**<0.001**
Thinks that chest compressions are most beneficial for outcome, n (%)	89 (55)	113 (59)	89 (58)	0.797	1.05 (0.84–1.32)	0.640
Is willing to perform CPR, n (%)	63 (40)	58 (31)	39 (26)	**0.027**	0.72 (0.57–0.92)	**0.008**
Knows that the defibrillator can save the patient’s life with eclectic shocks, n (%)	156 (98)	186 (98)	142 (93)	**0.034**	0.62 (0.33–1.14)	0.124
Knows that everybody is allowed to use an AED in case of CA, n (%)	90 (57)	122 (65)	47 (31)	**<0.001**	0.59 (0.47–0.75)	**<0.001**
Knows the colour of the standardized logo for AEDs, n (%)	100 (62)	87 (45)	64 (42)	**<0.001**	0.65 (0.52–0.82)	**<0.001**
Is willing to use an AED, n (%)	90 (57)	104 (54)	57 (37)	**0.001**	0.68 (0.54–0.85)	**0.001**

*p-value for significance of the regression model.

BLS = basic life support; AED = automated external defibrillator/defibrillation; CPR = cardiopulmonary resuscitation; CA = cardiac arrest.

## Discussion

To the best of our knowledge, the present analysis represents the largest survey on knowledge and awareness on both BLS and AED-use within the Austrian population in literature. We observed a poor level of education with regard to BLS attempts and the use of an AED device among the unselected Viennese community.

### Willingness to perform basic life support from an international perspective

Interestingly, while we found that more than 50 percent of participants would presume CA correctly and could properly initiate BLS attempts, only 33 percent reported that they were willing to perform CPR. Of note, the willingness to use an AED was significantly higher with 50 percent of all questioned persons. Our results are in line with an analysis from the Netherlands, that identified that only a minority of participants had sufficient education and willingness to use an AED device in case of OHCA—mirroring that more than half of the questioned persons had no knowledge on AEDs and were unwilling to use the device. Similar results also applied for a recent study from Great Britain. [12–13]

The reasons for this issue have widely been investigated and remain multifactorial. They include a lack of profound layperson willingness to perform BLS and apply an AED in public emergency situations, as well as a lack of knowledge of public AED locations. [13] This is also reflected by our results—only 50 percent of participants were able to recognize the standardized logo for an AED location.

In this regard, simple interventions are needed to ensure that devices are clearly and prominently marked in order to foster the visibility of devices in the community. Their visibility might significantly contribute to improve the AED awareness throughout society and consequently increase their use in case of emergency. Additionally, in the era of smartphones, a unified app for public AED locations might help to easily identify the nearest AED device and increase the use of public AEDs in case of OHCA. Moreover, misguided concerns over legal liabilities and poor levels of first-aid education among the western society strongly impact on both the awareness and willingness concerning BLS and AED-use within communities. [14] Therefore, despite many campaigns to improve public awareness on BLS and AED-use, it seems of utmost importance that BLS/AED curricula need to be tailored and adapted to their respective audience and might need to consider both age and gender specific aspects.

### Gender aspects in basic life support and AED-use

Interestingly, although the knowledge of the importance of BLS and the use of AEDs in case of OHCA was comparable between male and female participants, we observed a significantly lower willingness to initiate BLS attempts and use an AED among questioned female individuals. This issue has not been investigated in literature so far and poses a novel aspect in the field of education on BLS and AED-use. Considering those results it might be beneficial to adapt the content of first-aid programs with a specific focus on female needs. Especially encouraging female participants in both the administration of BLS and the use of an AED device in emergency situations might strongly impact on the willingness to attempt critical care efforts in real-life emergency care.

### The potential influence of the BLS providers’ age

After stratification into age-groups (<45years; 45-65years: >65years) we found that the youngest age-strata clearly demonstrated the best knowledge of BLS and AED use and the highest willingness to perform critical care attempts in case of witnessed OHCA. Interestingly, we observed a strongly decreasing level of knowledge and willingness for BLS assessment and AED-use with ingesting age. Our data give the impression of individuals younger than 45 years being more likely to provide BLS than their elderly counterparts. In this regard, many educational programs especially targeting the young community such as first-aid programs for school children were initiated in the past—these might now have impacted on awareness and knowledge in this age strata. On the other hand, the observations among the elderly community might be based on a fading memory of a once completed first-aid course many years ago. Therese findings may reflect a strong need of an enhanced initiation of both educational and training strategies as well as refreshment course programs tailored for the elderly individuals.

Of note, based on demographic changes within the next decades, the population older than 65 years will strongly increase in numbers. [15] Therefore it seems of utmost importance to elucidate concepts for BLS/AED curricula to fill the gap of knowledge especially in this age group.

The observed level of knowledge was far from excellent despite the fact that 81.4% of all participants completed a first-aid training. Similar results were presented by Brooks and co-workers. A potential explanation of the observed poor level of education might represent the fact that first-aid courses may have been completed several years back in many participants—considering that more than 68.2% of the study population are older than 45 years. This subsequently leads to a degree of forgetting in the middle-aged and elderly population and therefore not being as educated in BLS than their younger counterparts. Considering the strong benefit of a short and compulsory first-aid course on the awareness of BLS and AED-use, it seems as most effective to undertake educational efforts already in school children—this strategy might contribute to seeing all members of society be trained in BLS and AED-use and foster long-term knowledge. [16]

### Limitations

The major limitation of the present study represents the exclusion of non-German speaking persons, mirroring a potential selection bias. Moreover, since this analysis was conducted as an empiric population-based analysis, we can only presume that participants who stated they would initiate BLS attempts and use an AED in case of witnessed OHCA would actually do so in a real-life situation—this also accounts for vice versa. A further limitation might represent the fact that the time of competition of the last first-aid course was not assessed. However, considering local legal regulations, the majority of participants completed a first-aid course at least when obtaining driver’s license. Moreover, since medical education changed over the course of years—including BLS-training and first-aid courses—this might have an impact on our results, considering that younger participants and/or those having received training more recently would probably have better and more up-to-date knowledge on this specific topic than their elderly counterparts.

### Conclusion

Concluding our results, we found an overall poor knowledge and awareness in BLS and the use of AEDs among the Viennese population—indicating that only one third of participants was willing to initiate BLS attempts. Female individuals reported the lowest willingness to perform BLS and use an AED in case of OHCA. Similar results applied for participants older than 65 years, who presented the poorest knowledge and willingness to perform BLS and AED-use. Considering those findings, specially tailored programs to increase the awareness among both the female community and the elderly society need to be considered for future educational interventions.

## Supporting information

S1 File Questionnaire (German): Original German version of the used questionnaire. (PDF)

S2 File Questionnaire (English): English translation of the used questionnaire. (PDF)
