# Supplementary material for: Gender and age-specific aspects of awareness and knowledge in basic life support
Source: PLoS One. 2018 Jun 12;13(6):e0198918. doi: 10.1371/journal.pone.0198918 (PMC5997304; doi:10.1371/journal.pone.0198918)
Supplement: S1 File — (PDF) [file pone.0198918.s001.pdf]

Fragebogen

Teilnehmer ID: ....

Wissen zum BLS und Defibrillator

F1 Haben Sie schon einmal einen Erste-Hilfe Kurs besucht?

- Ja
- Nein

F2 Trauen Sie es sich zu, einen Menschen bei einem Atem-Kreislauf-Stillstand wiederzubeleben?

- Ja
- Nein
- Unsicher

F3 Ist es aus ihrer Sicht wichtig, dass Personen <u>ohne eine medizinische Ausbildung</u> eine Wiederbelebung bei einem Atem-Kreislauf-Stillstand durchführen können?

- Ja
- Nein
- Unsicher

F4 Würden Sie versuchen einen Fremden mit Atem-Kreislauf-Stillstand in der Öffentlichkeit wiederzubeleben?

- Ja
- Nein
- Unsicher

F5 Würden Sie an einem Fremden mit Atem-Kreislauf-Stillstand in der Öffentlichkeit einen Defibrillator anwenden?

- Ja
- Nein
- Unsicher

Ich stelle Ihnen nun einige Fragen zum Thema Atem-Kreislauf-Stillstand. Zu jeder Frage lese ich Ihnen vier Antwortmöglichkeiten vor. Bitte wählen Sie aus den vier Antwortmöglichkeiten die Antwortmöglichkeit aus, welche aus Ihrer Sicht richtig ist.

F6 Was macht man als nächstes, wenn ein Mensch auf Ansprechen und Schütteln der Schultern nicht reagiert?

- Lagerung in stabiler Seitenlage
- <u>Atemkontrolle</u>
- Lagerung mit erhöhten Beinen ("Schocklagerung")
- Die Mundhöhle auf Fremdkörper kontrollieren

F7 Was hilft einem Menschen mit Atem-Kreislauf-Stillstand am meisten?

- o <u>Herzdruckmassage</u>
- o Lagerung in stabiler Seitenlage
- o Beatmung
- o Lagerung mit erhöhten Beinen ("Schocklagerung")

F8 Wissen Sie wie das Gerät heißt, mit dem man einem Menschen mittels eines Stromimpulses (Schock) das Leben retten kann?

- o <u>Defibrillator / Defi</u>
- o EKG
- o Röntgengerät
- o Stethoskop

F9 Wissen Sie wer einen Defibrillator / Defi bedienen darf?

- o <u>Ausnahmslos jeder</u>
- o Nur Sanitäter und Ärzte
- o Ärzte mit Zusatzausbildung
- o Personen, die zumindest einen Erste-Hilfe Kurs besucht haben

F10 Wissen Sie welche Farbe das Defi-Zeichen hat?

- o <u>Grün</u>
- o Rot
- o Blau
- o Schwarz

Demographie

P1 Alter: ……….. Jahre

P2 Geschlecht

- o Mann
- o Frau

P3 Höchste abgeschlossene Schulbildung

- o Pflichtschule
- o Lehre, Fachschule ohne Matura
- o Matura
- o Universität, (Fach-) Hochschule

P4 Besitzen Sie die österreichische Staatsbürgerschaft?

- o Ja, seit der Geburt.
- o Ja, eingebürgert.
- o Nein
