# Supplementary material for: Gender and age-specific aspects of awareness and knowledge in basic life support
Source: PLoS One. 2018 Jun 12;13(6):e0198918. doi: 10.1371/journal.pone.0198918 (PMC5997304; doi:10.1371/journal.pone.0198918)
Supplement: S2 File — (PDF) [file pone.0198918.s002.pdf]

Questionnaire

Participant ID: ....

Knowledge in BLS and AED use

F1 Have you ever attended a first-aid course?

- Yes
- No

F2 Do you feel confident to perform resuscitation attempts on a person with cardiac arrest?

- Yes
- No
- Not sure

F3 Do you think it is important that people not having any medical background are able to perform resuscitative attempts?

- Yes
- No
- Not sure

F4 Would you perform resuscitative attempts on a foreign person in public that presents with cardiac arrest?

- Yes
- No
- Not sure

F5 Would you use a defibrillator on a foreign person in public that presents with cardiac arrest?

- Yes
- No
- Not sure

Hereinafter, I will ask some questions about cardiac arrest. For each question, four possible answers are given. Please note that only one answer is correct. Please select the right one.

F6 What is the next step if a person is unresponsive and does not react on shaking his shoulders?

- Place person in the recovery position
- <u>Check if person is breathing normally</u>
- Place person with elevated legs (shock position)
- Check mouth cavity for foreign objects

F7 What does a person with cardiac arrest benefit the most from?

- Chest compressions
- Place person in the recovery position
- Rescue breaths
- Place person with elevated legs (shock position)

F8 Do you know what the device is called by which you can save a person's life with an electric impulse (shock)?

- Defibrillator / AED
- ECG
- X-ray machine
- Stethoscope

F9 Do you know who is allowed to use an automated external defibrillator (AED)?

- Everyone
- Only paramedics and doctors
- Doctors with additional training
- Everyone who attended at least one first-aid course

F10 Do you know the colour of the sign that indicates the location of a defibrillator in public places?

- Green
- Red
- Blue
- Black

Demographics

P1 Age: ……….. years

P2 Gender

- Male
- Female

P3 Highest educational attainment

- Compulsory school
- High school
- School leaving examination
- University, Technical College

P4 Are you an Austrian Citizen?

- Yes, since birth
- Yes, naturalized
- No
